# Gold nanobipyramid-embedded ultrathin metal nanoframes for *in situ* monitoring catalytic reactions†

**DOI:** 10.1039/c9sc06475c

**Published:** 2020-02-26

**Authors:** Xingzhong Zhu, Juan Xu, Han Zhang, Ximin Cui, Yanzhen Guo, Si Cheng, Caixia Kan, Jianfang Wang

**Affiliations:** College of Science, Nanjing University of Aeronautics and Astronautics Nanjing 210016 China cxkan@nuaa.edu.cn; Department of Physics, The Chinese University of Hong Kong Shatin Hong Kong SAR China jfwang@phy.cuhk.edu.hk; College of Chemistry, Chemical Engineering and Materials Science, Soochow University Suzhou 215021 China chengsi@suda.edu.cn

## Abstract

Metal nanoframes, especially ultrathin ones, with excellent plasmonic properties are synthetically interesting and highly attractive. Herein we report on the synthesis of Au nanobipyramid-embedded ultrathin metal nanoframes with one of the plasmon modes very similar to that of the Au nanobipyramids. The synthesis is mediated by silver coating on Au nanobipyramids. The excellent plasmonic properties of the Au nanobipyramid-embedded ultrathin metal nanoframes are ascribed to the little influence of the ultrathin metal nanoframes on the Au nanobipyramids, as verified by electrodynamic simulations. The increase in the amount of the added metal atoms changes the nanostructure from the nanoframe to a nanocage shape. The method has also been successfully applied to (Au nanobipyramid)@Ag nanorods with different lengths and Au nanobipyramids with different longitudinal dipolar plasmon wavelengths, suggesting the generality of our approach. We have further shown that the Au nanobipyramid-embedded ultrathin metal nanoframes possess an excellent surface-enhanced Raman scattering and outstanding *in situ* reaction probing performance. Our study opens up a route for the construction of plasmonic ultrathin metal nanoframes based on Au nanobipyramids for plasmon-enabled applications.

## Abstract 2

Ultrathin gold, palladium and platinum nanoframes are synthesized for monitoring reactions. Each nanoframe contains a gold nanobipyramid at its center.

## Graphical abstract

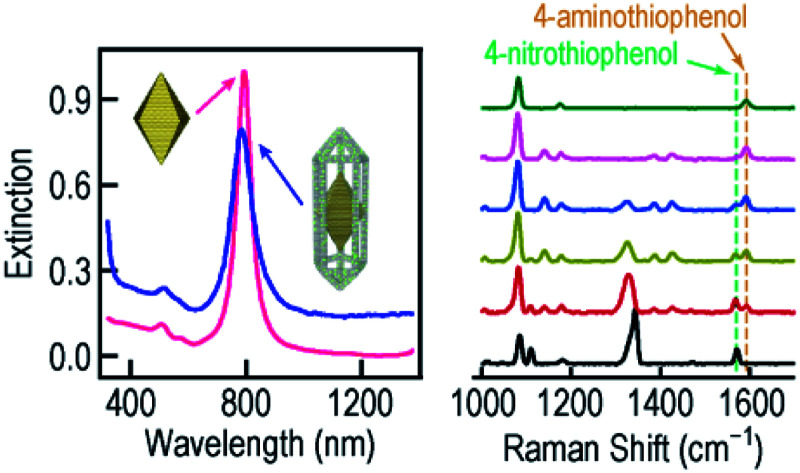

## Introduction

Metal nanostructures have attracted enormous interest in a wide range of areas, such as waveguides,^1^ optics,^2,3^ solar energy harvesting,^4^ photocatalysis,^5,6^ sensing^7,8^ and biomedicine.^9,10^ The physicochemical properties of metal nanostructures are well known to be strongly correlated with their size and shape. As a result, tremendous efforts have been devoted to the synthesis of metal nanostructures with a myriad of different shapes, including nanospheres,^11^ nanorods,^12^ nanocubes,^13^ nanoboxes,^14^ nanowires,^15^ nanocages^16^ and nanoplates.^17^ Among them, metal nanoframes have recently received much attention for a number of applications owing to their highly open structure and high specific surface area.^18–23^ Ultrathin metal nanoframes, with the frame thinned to the sub-5 nm or even sub-2 nm scale, have been intensively studied for different applications.^24–26^ For example, the synthesis of azobenzene from aniline catalyzed by ultrathin Au–Ag nanoframes gives a yield of 94%, higher than that of 31% when the same amount of Au–Ag nanoparticles is used.^27^ Ultrathin Pt_3_Ni nanoframes offer a 36-fold enhancement in mass activity for the oxygen reduction reaction.^28^ Ultrathin Au–Pt_3_Ni nanoframes show a higher initial turnover frequency than Pt_3_Ni frames and almost 100% selectivity towards 4-aminobenzaldehyde for the reduction of 4-nitrobenzaldehyde.^29^

Plasmon is an important property of metal nanostructures. It has enabled widespread potential applications in photochemistry, photomagnetism, photoelectricity and photobiology. However, there have been rare reports on the plasmonic properties of ultrathin metal nanoframes no matter what metal element or composition the nanoframes are made of. On the one hand, mono- and multi-metallic ultrathin nanoframes made of metals (Pd, Pt, Rh, and Ru) that are not good plasmonic materials lack distinct plasmon resonances because of the large imaginary parts of the dielectric functions of these metals.^30–33^ On the other hand, the plasmon wavelengths of ultrathin metal nanoframes made of the plasmonic metals (Au, Ag, and Cu) are beyond 2000 nm, usually in the mid-infrared region, and have relatively weak intensities. In addition, the plasmon peaks are often severely broadened inhomogeneously and become unobservable.^34–37^ These drawbacks have severely hindered the applications of plasmonic ultrathin metal nanoframes. The development of ultrathin metal nanoframes with excellent plasmonic properties has therefore still remained challenging and highly desirable for the extension of ultrathin metal nanoframes to various plasmon-enabled applications in areas such as optics,^3^ photocatalysis,^6^ sensing,^38^ imaging^39^ and drug release.^40^

In this work, we demonstrate the synthesis of ultrathin metal nanoframes with the assistance of Ag. Each metal nanoframe encapsulates a gold nanobipyramid (NBP). The produced Au NBP-embedded ultrathin metal nanoframes exhibit several plasmon resonance modes, with one of them being very similar to that of the core Au NBP, including the plasmon wavelength and intensity. The construction of the metal nanoframe around a gold NBP has nearly no effect on the plasmonic properties of the latter. The origin of the unique plasmonic properties of the Au NBP-embedded ultrathin metal nanoframes has been systematically examined as a function of the structure and composition. The supplied metal atoms are found to enlarge from the corners and edges to the surfaces of the pre-prepared (Au NBP)@Ag nanorods with the increase of the metal precursor amount. The generality of this method is verified by the successful construction of the ultrathin nanoframes with different lengths on Au NBPs, as well as the ultrathin nanoframes on Au NBPs with different longitudinal dipolar plasmon wavelengths. Moreover, the superior surface-enhanced Raman scattering (SERS) probe characteristics of the Au NBP-embedded ultrathin metal nanoframes are demonstrated by *in situ* SERS-monitoring metal-catalyzed reactions.

## Results and discussion

**Fig. 1:**
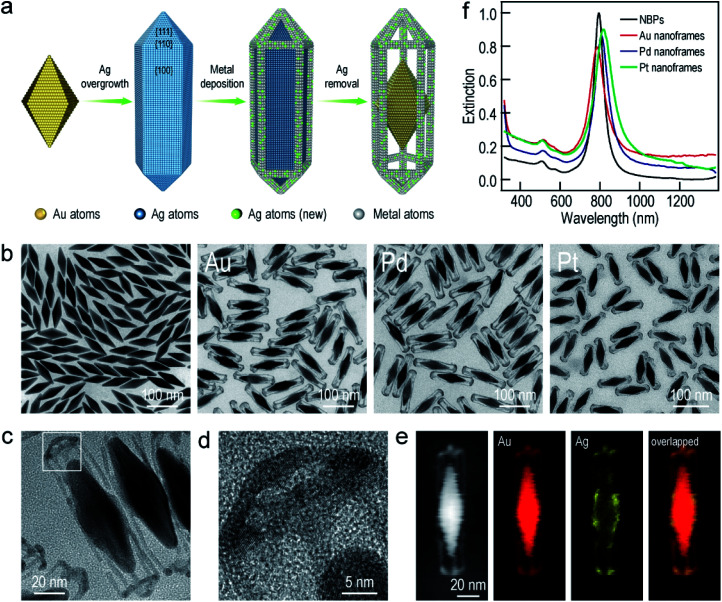
Au NBP-embedded ultrathin metal (Au, Pd, and Pt) nanoframes. (a) Schematic illustrating the synthetic process. (b) TEM images of the Au NBP sample and the Au NBP-embedded metal (Au, Pd, and Pt) nanoframe samples. (c) TEM image of several Au NBP-embedded Au nanoframes. (d) HRTEM image recorded in the region indicated with the white box in (c). (e) HAADF-STEM and elemental mapping images of a single Au NBP-embedded ultrathin Au nanoframe. The elemental mapping images have the same scale bars as the HAADF-STEM image. (f) Extinction spectra of the Au NBP sample and the Au NBP-embedded metal (Au, Pd, and Pt) nanoframe samples.

The Au NBP samples were prepared through a stepwise combination of seed-mediated growth and subsequent depletion force-induced purification.^41^ The overgrowth of Ag on the Au NBPs, the deposition of the metals (Au, Pd, and Pt), and the subsequent removal of Ag followed the procedures reported in previous studies.^42,43^ The surfactant and pH were found to be crucial for the anisotropic deposition of a second metal on the pre-grown Ag nanocrystals.^37,44^Fig. 1a illustrates schematically the experimental procedure for the synthesis of the Au NBP-embedded ultrathin metal nanoframes. The overgrowth of Ag on the Au NBPs causes the transformation from the bipyramid shape to a pentagonal prism shape to give the (Au NBP)@Ag nanorods.^42^ The deposition of ultrathin metal nanoframes is realized through the synergetic action of galvanic replacement and co-reduction of metal ions. Because Br^−^ ions derived from cetyltrimethylammonium bromide (CTAB) can selectively bind to the side facets, that is, the {100} facets, on the (Au NBP)@Ag nanorods, we argue that these sites will be preferentially activated toward oxidation. Upon the addition of a metal precursor, the {100} facets can act as an anode for the initiation of the galvanic replacement between Ag and the metal complex precursor. The released Ag^+^ ions should stay soluble by complexing with Br^−^ ions to form AgBr_2_^−^. At the same time, both AgBr_2_^−^ and the metal complex precursor can be reduced by ascorbic acid to generate Ag and the desired metal atoms, followed by their deposition on the (Au NBP)@Ag nanorods.^45^ Because the side facets are involved in the galvanic replacement reaction, the co-deposition of Ag and the desired metal atoms is automatically directed and confined to the {110} facets at the edges and corners because of their higher free energies.^37^ The Ag atoms are increasingly carved away from the side facets, leading to the formation of (Au NBP)@Ag@AgM (M = Au, Pd, Pt) nanoframes. Upon the etching of the pure Ag segments in the core with H_2_O_2_, the (Au NBP)@Ag@AgM nanoframes are transformed into (Au NBP)@AgM nanoframes.

Transmission electron microscopy (TEM) imaging of the Au NBP sample and the Au NBP-embedded metal nanoframes reveals the uniform morphologies and narrow size distributions of all samples (Fig. 1b). The purified Au NBP sample has an average length of 98 ± 3 nm and waist width of 28 ± 1 nm. Each nanostructure contains one Au NBP at the center, with an ultrathin metal nanoframe surrounding the NBP core. The ultrathin metal nanoframes possess a three-dimensional geometric shape with a virtually pentagonal morphology. Such a deposition behavior is not affected by the type of the added metal precursor. The difference in the morphology for the Pt nanoframes can be attributed to the much higher bonding energy of Pt–Pt (307 kJ mol^−1^) than that of Pt–Ag (218 kJ mol^−1^). In contrast, the bonding energies of Pd–Pd (100 kJ mol^−1^) and Au–Au (226 kJ mol^−1^) are smaller than those of Pd–Ag (137 kJ mol^−1^) and Au–Ag (229 kJ mol^−1^), respectively, favoring the formation of Pd–Ag and Au–Ag alloys rather than the separation into the different phases.

In order to find out the composition of the ultrathin metal nanoframes around the Au NBPs, we performed high-angle annular dark-field scanning transmission electron microscopy (HAADF-STEM) imaging and elemental mapping (Fig. 1c–e). High-resolution transmission electron microscopy (HRTEM) imaging (Fig. 1c and d) reveals that most of the Ag atoms are selectively removed, leaving behind the pentagonal nanoframes with a unique open structure. The ridges in the nanoframes are only 2–3 nm in thickness. Elemental mapping clearly shows the presence of Au and Ag atoms on the nanoframes, while the Au atoms located dominantly at the center maintain the bipyramidal shape. The metal components of the nanoframes are therefore Au-enriched Au–Ag alloy, as evidenced by the complete overlap of the elemental distributions of Au and Ag on the elemental maps.^46^ The residual Ag atoms at the gaps between the nanoframes and the middle of the Au NBPs can be ascribed to the weak oxidation ability of H_2_O_2_ in the etching process. They connect the Au NBPs with the nanoframes to keep the Au NBP fixed in the center of the metal nanoframe. The results for the Au NBP-embedded Pd and Pt nanoframes further reveal that the nanoframes are composed of metal (Au, Pd, Pt)–Ag alloys (Fig. S1 and S2†), which verifies the schematic shown in Fig. 1a. For simplicity, the nanoframes are named as the Au, Pd and Pt ones according to the salt precursor used in the synthesis. The deposition of the ultrathin metal nanoframes on the Au NBPs causes a slight shift of the longitudinal plasmon peak, which is accompanied by slight peak broadening and intensity reduction (Fig. 1f). For the longitudinal plasmon peaks, the full widths at half maximum (FWHMs) are 62 nm, 87 nm, 80 nm and 107 nm for the Au NBPs, and Au NBP-embedded ultrathin Au, Pd and Pt nanoframes, respectively. The transverse plasmon peak, however, shows slight shifts. The peak broadening and intensity reduction arise mainly from the increase of the size distribution upon the growth of the ultrathin metal nanoframes, because the starting Au NBPs are highly uniform in shape and size.^41,47^

**Fig. 2:**
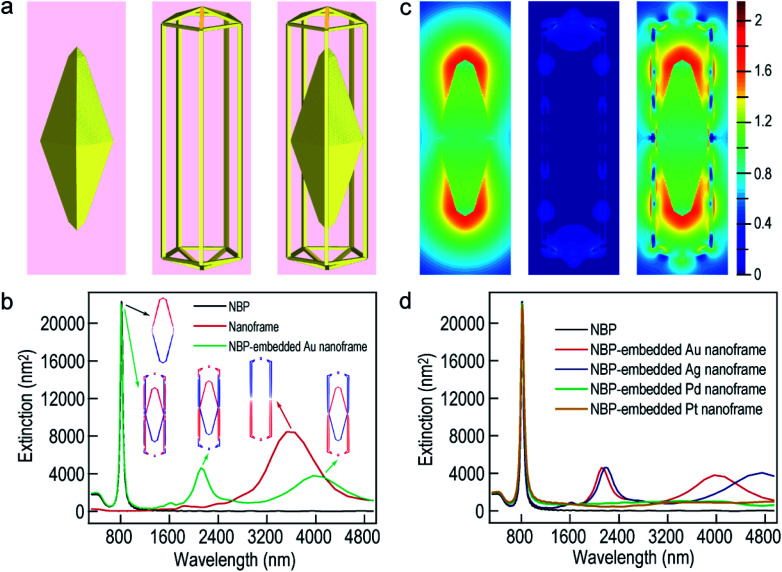
FDTD simulations. (a) Schematics of the simulation models. (b) Simulated extinction spectra of the Au NBP, Au nanoframe, and Au NBP-embedded Au nanoframe. The insets are the charge distribution contours calculated at the peak wavelengths under the longitudinal excitation. If the five side ridges of the nanoframe are labeled as 1–5 consecutively, the cross-section for showing the charge distribution contours is determined by ridges 1 and 3, and it is perpendicular to the propagation direction of the excitation light. The red and blue colors represent positive and negative charges, respectively. (c) Simulated electric field enhancement contours under the longitudinal excitation at 815 nm for the Au NBP (left), Au nanoframe (middle), and Au NBP-embedded Au nanoframe (right), respectively. The field enhancement contours are drawn at the logarithmic scale. (d) Simulated extinction spectra of the Au NBP and the Au NBP-embedded Au, Ag, Pd and Pt nanoframes.

To further understand the nearly identical plasmon resonance before and after the deposition of the ultrathin metal nanoframes, finite-difference time-domain (FDTD) simulations were performed to ascertain the plasmonic properties of the Au NBP-embedded ultrathin Au nanoframes (Fig. 2). The nanoframes were assumed to be made of pure Au for simplification, which has been shown to be reasonable in the simulation of the plasmonic properties of metal alloy nanostructures.^48^ The geometrical models employed in the simulations were set according to the shapes observed in the TEM images. The length and waist width of the penta-twinned Au NBP were set to be 98 nm and 31 nm, respectively (Fig. 2a). The ultrathin Au nanoframe has pentagonal symmetry with the ridge being 2 nm × 2 nm × 119 nm. The nanoframe is in contact with the middle corners of the Au NBP for the Au NBP-embedded Au nanoframe. The excitation light direction was perpendicular to the length axis, with the electric field polarized along the length axis. As a result, only the longitudinal plasmon modes were excited. The Au NBP has a longitudinal plasmon peak at 815 nm, which can be unambiguously attributed to the dipolar mode (Fig. 2b).^49,50^ For the ultrathin Au nanoframe without the Au NBP, the charges on the inner and outer surfaces oscillate in phase, producing a dipolar bonding mode at 3523 nm according to the plasmon hybridization model.^51^ The antibonding mode cannot be effectively excited by far-field light owing to a nearly zero net dipole moment. Interestingly, when a gold NBP is embedded in the ultrathin Au nanoframe, both of the antibonding and bonding dipolar modes of the nanoframe can be excited. They couple with the dipolar mode of the Au NBP, resulting in two peaks at 808 nm and 4133 nm, respectively. The presence of the Au NBP at the center also induces the excitation of an octapolar mode of the nanoframe, which is usually a dark mode. A new peak at 2116 nm can therefore be assigned to the combination of the NBP dipolar and the nanoframe octapolar modes. Simulations were further conducted to clarify the nature of the plasmon resonances of the Au NBP-embedded Au nanoframe. The structural changes at the ends of the nanoframe were found to have little effect on the plasmon resonances of the Au NBP-embedded Au nanoframe (Fig. S3†), indicating that the interaction mainly takes place between the side ridges of the nanoframe and the Au NBP. As shown in Fig. S4,† the two low-energy peaks of the ultrathin Au nanoframes in the mid-infrared region exhibit gradual redshifts as the width of the ridges is decreased or the length of the side ridges is increased, while the highest-energy plasmon peak remains almost unchanged at ∼800 nm. The transmission measurements in the mid-infrared region show that the two low-energy peaks observed in the simulations cannot be detected at the ensemble level, which can be ascribed to the inherent low intensities of the two peaks and the inhomogeneous distributions in shape and size (Fig. S5†). On the other hand, the simulated results are generally in good agreement with the experimental ones for the longitudinal plasmon wavelength in the visible region. The longitudinal plasmon mode of the Au NBP causes a maximal electric field enhancement at the two ends, while the sole Au nanoframe without the Au NBP exhibits weak enhancement (Fig. 2c). The largest electric field enhancement is still located at the two ends of the Au NBP under longitudinal excitation for the Au NBP-embedded Au nanoframe, which is also the case for the Au NBP-embedded Pd and Pt nanoframes (Fig. S6†). However, the nanoframe segments that are adjacent to the two ends of the Au NBP also show remarkable electric field enhancement when the Au NBP is embedded.

We also performed FDTD simulations by replacing the ultrathin Au nanoframes around the Au NBP with Ag, Pd and Pt nanoframes. Intriguingly, the main plasmon wavelengths of the four types of Au NBP-embedded ultrathin metal nanoframes remain approximately the same as that of the Au NBP, with the Pd and Pt ones showing no peaks in the mid-infrared region (Fig. 2d). These simulation results unambiguously indicate that the main plasmon peak observed in the Au NBP-embedded metal nanoframes is mainly determined by the Au NBP while the other peaks in the mid-infrared region are associated with the metal nanoframe. The type of ultrathin metal nanoframe around the Au NBP plays a minor role in the main plasmon peak of the Au NBP-embedded metal nanoframes. The growth of the ultrathin metal nanoframes around the Au NBPs provides a powerful means for the construction of plasmonic ultrathin nanoframes based on the excellent plasmonic properties of Au NBPs.

**Fig. 3:**
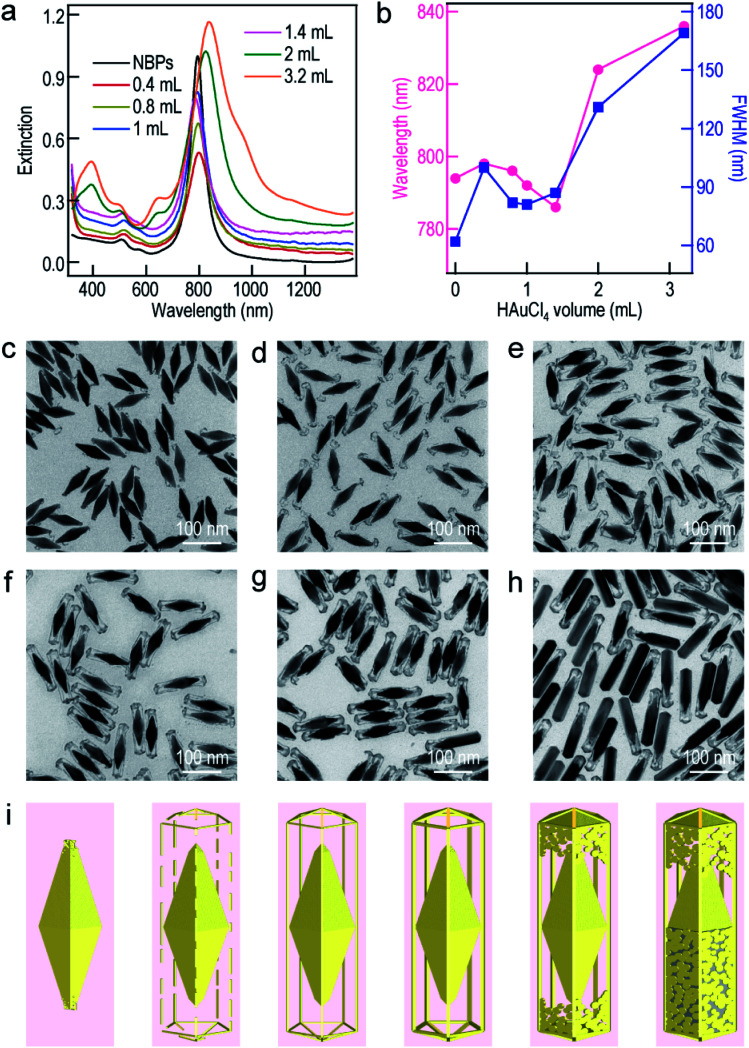
Preparation of the Au NBP-embedded nanostructures using HAuCl_4_ at different amounts. (a) Extinction spectra of the Au NBP sample and the Au NBP-embedded nanostructure samples produced with HAuCl_4_ at different amounts. (b) Variations of the longitudinal plasmon wavelength and FWHM as a function of the volume of added HAuCl_4_. (c–h) TEM images of the produced Au NBP-embedded nanostructure samples. (i) Schematics illustrating the morphological evolution of the Au NBP-embedded nanostructures with different amounts of HAuCl_4_.

To further understand the growth mechanism of the ultrathin metal nanoframes on the Au NBPs, the Au nanoframes were systematically studied as a representative example. The surfactant concentration was found to be crucial for the successful preparation of the Au NBP-embedded Au nanoframes during the Ag etching process (Fig. S7†). As the CTAB concentration is increased, the longitudinal plasmon peak gradually blueshifts and becomes steady at a point of 1.5 mM. This result arises mainly from the need for sufficient Br^−^ to form AgBr_2_^−^ in the etching process.^52^ The time-dependent etching experiments carried out on the Au NBP-embedded Au nanoframes show that the removal of Ag is almost completed after the first 30 min (Fig. S8†). With the increase in the supplied HAuCl_4_ amount, the longitudinal plasmon resonance of the Au NBP-embedded nanostructures first blueshifts and then redshifts. The turning point is located at 1.4 mL (Fig. 3a). The transverse plasmon peak, however, shows very small shifts before the turning point. When the HAuCl_4_ volume is more than 1.4 mL, two new peaks appear at ∼400 nm and ∼650 nm. They can be attributed to the transverse multipolar plasmon mode and the higher-order longitudinal plasmon mode of the Ag nanostructures, respectively, indicating the existence of residual Ag atoms after the etching process. The longitudinal plasmon wavelengths and FWHMs are plotted in Fig. 3b as a function of the added HAuCl_4_ volume. The FWHMs display an almost identical variation trend to the plasmon wavelengths. The Au NBP-embedded nanostructures grown with HAuCl_4_ at different amounts were imaged under TEM (Fig. 3c–h). The imaging results show that the deposited Au atoms have different morphologies around the Au NBPs. When the volume is too small (0.4 mL), the deposited Au atoms are insufficient to interconnect to form a framework. They form small particles upon the dissolution of the Ag template. The attachment of the Au nanoparticles on the Au NBPs induces the redshift and peak broadening of the plasmon resonance. The Au frames are structurally fragile and disintegrate into smaller rod fragments at 0.8 mL. Because of the high selectivity for the deposition at the edges and corners, with progressive increases in the amount of Au, a point (1.0 mL and 1.4 mL) is reached where the formed Au frames are robust and maintained after Ag dissolution (Fig. 3e and f). As the volume of HAuCl_4_ is further increased, Ag dissolution gives porous shells with gold remaining at both the facets and edges (Fig. 3g and h). Even Ag cannot be etched when the volume of HAuCl_4_ reaches 3.2 mL. The appearance of the porous shells (Fig. S9†) can be ascribed to the surface diffusion of Au atoms.^36,53^ The HAuCl_4_ amount-dependent shape evolution of the Au NBP-embedded nanostructures is schematically illustrated in Fig. 3i. The overall shape variation trend is similar to that of the TEM images shown in Fig. 3c–h.

**Fig. 4:**
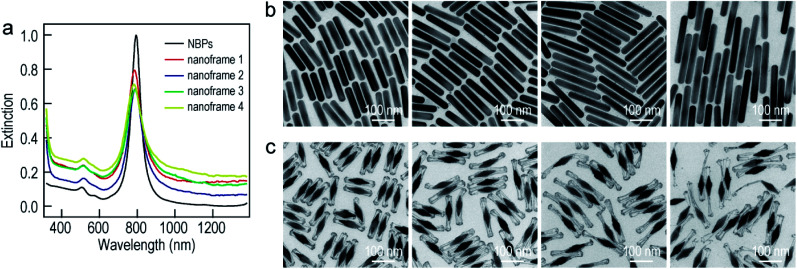
Au NBP-embedded ultrathin Au nanoframes with different lengths. (a) Extinction spectra of the Au NBP sample and the Au NBP-embedded ultrathin Au nanoframe samples with different lengths. (b) TEM images of the (Au NBP)@Ag nanorod samples grown with 250 μL, 350 μL, 450 μL and 500 μL of AgNO_3_ (0.01 M), respectively. (c) TEM images of the corresponding Au NBP-embedded ultrathin Au nanoframe samples, respectively.

To improve the generality of our approach and further verify the growth mechanism, we investigated the growth of the ultrathin Au nanoframes with length control on the Au NBPs (Fig. 4). The length of the ultrathin Au nanoframes was found to be dependent on that of the pre-prepared (Au NBP)@Ag nanorods. Fig. 4a displays the extinction spectra of the Au NBP sample and four ultrathin Au nanoframe samples with varied lengths. The longitudinal plasmon wavelengths of the ultrathin Au nanoframe samples with different lengths are similar to that of the Au NBPs. The peak broadening and intensity reduction with the increase in the length of the nanoframes might arise from three possible reasons: the increase in the size distribution after the deposition of the ultrathin Au nanoframes, various distortions of the Au nanoframes, and additional Au atoms deposited on the surface of the Au NBPs during the growth of the Au nanoframes (Fig. S10†). The TEM images of the (Au NBP)@Ag nanorod samples and their corresponding Au NBP-embedded Au nanoframe samples are shown in Fig. 4b and c, respectively. The (Au NBP)@Ag nanorods are uniform in diameter along the length direction, with their average diameters all being 30 ± 1 nm. The average lengths are 136 ± 5 nm, 158 ± 7 nm, 203 ± 11 nm and 248 ± 19 nm, respectively. The length of the ultrathin Au nanoframes increases as the corresponding (Au NBP)@Ag nanorods become longer. The ultrathin Au nanoframes start to lose their continuity and structural integrity as they become very long.

**Fig. 5:**
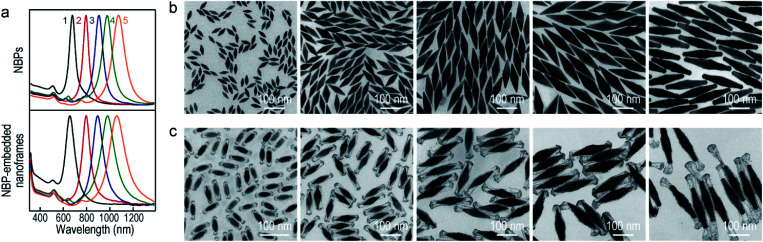
Au NBP-embedded ultrathin Au nanoframes with different plasmon wavelengths. (a) Normalized extinction spectra of the Au NBP samples (top) and the corresponding ultrathin Au nanoframe samples (bottom). (b) TEM images of the Au NBP samples with their extinction spectra labeled as 1–5 in (a). (c) TEM images of the corresponding ultrathin Au nanoframe samples. The supplied volumes of AgNO_3_ (0.01 M) are 350 μL, 250 μL, 300 μL, 300 μL, and 250 μL, and those of HAuCl_4_ (0.1 mM) are 2.2 mL, 1.4 mL, 2.4 mL, 2.4 mL and 3.5 mL, respectively.

To produce Au NBP-embedded ultrathin Au nanoframes with different plasmon wavelengths, four Au NBP samples with longitudinal dipolar plasmon wavelengths at 679 nm, 906 nm, 979 nm and 1074 nm, respectively, were synthesized for the growth of ultrathin Au nanoframes. Fig. 5 displays the extinction spectra and TEM images of the five Au NBP samples (including the 794 nm sample shown in Fig. 1), together with the corresponding Au NBP-embedded Au nanoframe samples. The longitudinal plasmon wavelengths of the ultrathin Au nanoframe samples 1–5 are 658 nm, 796 nm, 894 nm, 980 nm and 1060 nm, respectively. The plasmon wavelengths of all of the five ultrathin nanoframe samples are similar to those of the starting Au NBP samples (Fig. 5a). The as-prepared Au NBPs possess uniform sizes and shapes, with their average lengths/waist widths determined by TEM imaging (Fig. 5b) to be 50 ± 2 nm/18 ± 1 nm, 98 ± 3 nm/28 ± 1 nm, 154 ± 5 nm/41 ± 2 nm, 195 ± 8 nm/49 ± 3 nm and 206 ± 10 nm/41 ± 2 nm, respectively. The prepared ultrathin Au nanoframes encapsulate the Au NBPs, irrespective of the sizes of the Au NBPs. For samples 1–3, the ultrathin Au nanoframes all show a pentagonal open structure with relatively dense ends, similar to that for the case of the 794 nm Au NBPs (Fig. 1). For samples 4 and 5, the ultrathin Au nanoframes are too long to sustain the largely hollow framework (Fig. S11†), which can be improved by slightly increasing the amount of HAuCl_4_ to form an ultrathin porous Au shell (Fig. 5c). The successful variation of the longitudinal plasmon wavelength spanning from the visible to the near-infrared region by judiciously choosing the starting Au NBPs makes our approach very attractive in the development of plasmonic applications by use of the ultrathin metal nanoframes.

**Fig. 6:**
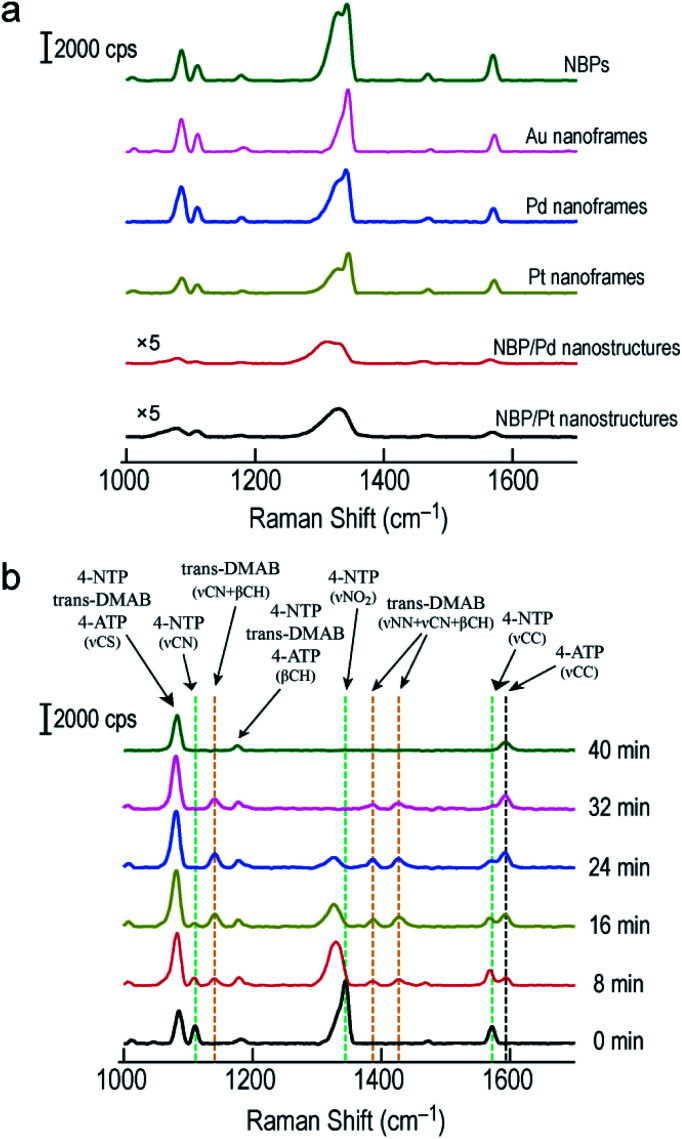
SERS measurements on the Au NBPs and Au NBP-derived nanostructures. (a) SERS spectra collected from 4-NTP adsorbed on the Au NBPs, Au NBP-embedded ultrathin metal (Au, Pd, Pt) nanoframes and (Au NBP)/(Pd, Pt) nanostructures under excitation at 785 nm. (b) Time-dependent SERS spectra collected during the reduction of 4-NTP by NaBH_4_ under excitation at 785 nm. The reaction was catalyzed by the ultrathin Au nanoframes shown in Fig. 1b.

SERS has become a powerful vibrational spectroscopic technique during the last few decades. The number of its applications in the chemical, materials, and in particular life sciences is rapidly increasing.^54–56^ The SERS performance has been found to be strongly affected by plasmonic properties. We therefore employed the Raman signal of 4-nitrothiophenol (4-NTP) functionalized on the surface of the different nanostructures to reveal the SERS performance of the Au NBP-embedded ultrathin metal nanoframes in comparison with that of the Au NBPs. Apart from the three types of ultrathin metal nanoframes, (Au NBP)/Pd and (Au NBP)/Pt nanostructures were also prepared for comparison (Fig. S12†). The deposition of Pd and Pt nanoparticles on the Au NBPs induces a redshift of 14 nm and 28 nm, respectively. The peak broadening and intensity reduction originate mainly from the inhomogeneous size distribution and the plasmon damping caused by the large imaginary parts of the Pd and Pt dielectric functions. TEM imaging reveals that the produced (Au NBP)/Pd and (Au NBP)/Pt nanostructures are uniform in both morphology and size. Each Au NBP core is covered with discrete Pd or Pt nanoparticles, whose sizes are 2–4 nm. In order to facilitate the comparison of the SERS intensities and the subsequent catalytic reactions, the particle concentrations of the Au NBP, Au NBP-embedded metal nanoframe and (Au NBP)/(Pd, Pt) nanostructure samples were adjusted to be identical, which was verified by inductively coupled plasma optical emission spectrometry (ICP-OES) measurements. The Au, Ag, Pd and Pt concentrations in the solutions were determined according to the calibration lines acquired in advance from standard Au, Ag, Pd and Pt solutions (Fig. S13†). The obtained Au, Ag, Pd and Pt concentrations for the six samples are listed in Table S1.† The Au concentrations of the Au NBP, Pd nanoframe, Pt nanoframe and (Au NBP)/(Pd, Pt) nanostructure samples were determined to be 6.9 mg L^−1^, while that of the Au nanoframe sample was 8.1 mg L^−1^. The Au concentration in the Au nanoframe sample is 17% larger. The corresponding value calculated from the simulation models is 21%. This similarity confirms the same particle concentration for the six samples. The respective amounts of the Pd and Pt atoms in the ultrathin Pd, Pt nanoframe and (Au NBP)/(Pd, Pt) nanostructure samples are almost the same. Such judicious adjustment makes the comparison among the six samples meaningful. Fig. S14† shows the SERS spectra collected from 4-NTP adsorbed on the ultrathin metal nanoframes with excitation at 514 nm and 633 nm, respectively. The ultrathin metal nanoframes show no Raman peaks under excitation at 514 nm. They show a single weak peak under excitation at 633 nm. These results can be attributed to the large spectral separation between the plasmon peaks (see Fig. 1f and S12a† for the extinction spectra of the six samples) and the excitation laser wavelengths. By changing the excitation laser wavelength to 785 nm, which is very close to the plasmon peaks of the six samples, the ultrathin metal nanoframes exhibit comparable SERS signals to those of the Au NBPs, while the (Au NBP)/Pd and (Au NBP)/Pt nanostructures give weaker SERS signals (Fig. 6a). Based on a reported protocol,^3,57^ we employed the peak at 1572 cm^−1^ to calculate the SERS enhancement factors (EFs, Table S2†). During the determination of the numbers of the probed molecules, we assumed that the surface was covered by a complete monolayer of 4-NTP molecules. The determined EFs for the Au NBPs, and the ultrathin Au, Pd and Pt nanoframes are (12 ± 3) × 10^7^, (3.3 ± 0.3) × 10^7^, (2.6 ± 0.2) × 10^7^ and (2.4 ± 0.2) × 10^7^, respectively. In comparison, the EFs are only (2.6 ± 1.3) × 10^6^ and (6.3 ± 1.5) × 10^6^ for the (Au NBP)/Pd and (Au NBP)/Pt nanostructures, respectively. The stronger SERS signals for the nanoframes should result from the lower plasmon damping due to the frame structure than that for the nanostructures with Pd or Pt nanoparticles deposited all over the surface of the Au NBP. As a result, the open frame structure makes the Au NBP-embedded ultrathin metal nanoframes an attractive candidate for SERS-related applications.

By combining the good SERS performance of the Au NBP-embedded metal nanoframes with the catalytic activity of the metal atoms on the nanoframes, we further demonstrated a unique SERS probe for *in situ* monitoring the reduction of 4-NTP to 4-aminothiophenol (4-ATP) by NaBH_4_ in the Au NBP-embedded Au nanoframe solutions (Fig. 6b). Specifically, the ultrathin Au nanoframes were mixed with 4-NTP solution (0.1 mM). 4-NTP was self-assembled as a monolayer on the surface of the ultrathin Au nanoframes. Upon the introduction of NaBH_4_ to initiate the catalytic reaction, we collected SERS spectra at different time points under excitation at 785 nm. At 0 min, the SERS spectrum of 4-NTP shows three characteristic vibrational bands (marked by the green dashed lines) at 1110 cm^−1^ (C–N stretching, ν_CN_), 1344 cm^−1^ (O–N–O stretching, ν_NO_2__), and 1572 cm^−1^ (C–C stretching on the phenyl ring, ν_CC_), respectively.^58,59^ At 8 min, the ν_NO_2__ vibration of 4-NTP redshifts from 1344 to 1329 cm^−1^, together with a slight decrease in intensity. The intensities of ν_CN_ and ν_CC_ decrease while their peak positions are essentially unchanged. On the other hand, four new bands emerge in the SERS spectrum. Specifically, the band at 1592 cm^−1^ can be assigned to the ν_CC_ mode of 4-ATP (marked by the black dashed line) and the other three bands at 1141 cm^−1^, 1388 cm^−1^ and 1427 cm^−1^ can be assigned to ν_CN_ + β_CH_, ν_NN_ + ν_CN_ and ν_NN_ + β_CH_ of *trans*-4,4′-dimercaptoazobenzene (*trans*-DMAB, marked by the orange dashed line), respectively.^60,61^ As the reaction progresses to 16 min and then 24 min, the ν_NO_2__ mode of 4-NTP (at 1329 cm^−1^) starts to decrease in intensity, together with a slight redshift in the peak position. The ν_CC_ mode of 4-NTP (at 1572 cm^−1^) also gradually decreases in intensity while that of 4-ATP (at 1592 cm^−1^) continuously increases in intensity. The three bands of *trans*-DMAB show an increase in intensity. At 32 min, the bands of 4-NTP eventually disappear, with those of *trans*-DMAB starting to decrease. At 40 min, the bands associated with 4-NTP and *trans*-DMAB all disappear. The three remaining peaks can be assigned to the ν_CS_, β_CH_ and ν_CC_ modes of 4-ATP, respectively. We also note that the peaks at 1085 cm^−1^ and 1181 cm^−1^ can be assigned to 4-NTP, *trans*-DMAB, or 4-ATP. They are relatively stable in all SERS spectra during the course of the reaction. The Au nanoframes were found to maintain the frame structure after the *in situ* monitoring of the catalytic reaction (Fig. S15†), suggesting the high stability of the ultrathin nanoframes during the reaction. The SERS spectra remained unchanged as a function of time in the control experiments performed with the Au NBPs and (Au NBP)@Ag nanorods under similar conditions (Fig. S16†), suggesting that Au and Ag intrinsically have no or a very low catalytic activity for this reaction. This result indicates that the catalytic activity has totally originated from the ultrathin Au nanoframes due to their large specific surface area and abundant highly active sites. The stronger SERS signals of the Au NBPs than those of the (Au NBP)@Ag nanorods can be ascribed to the reasons including the stronger Au–S bond, the higher resistance to oxidation, the sharper tips, and the closer plasmon peak to the excitation wavelength for the Au NBPs. The replacement of the ultrathin Au nanoframes with the ultrathin Pd and Pt nanoframes gave similar results, except that the peaks of *trans*-DMAB cannot be observed (Fig. S17†). The comparison between the Au NBP-embedded Pd nanoframes and the (Au NBP)/Pd nanostructures, as well as the Pt-related ones, indicates that the ultrathin metal nanoframes are better than their corresponding (Au NBP)/metal nanostructures as a probe for monitoring catalytic reactions by SERS due to the unique plasmonic properties of the former. On the other hand, as discussed above, the nanoframe segments that are adjacent to the two ends of the Au NBP exhibit remarkable electric field enhancement because of the presence of the Au NBP. In consideration of monitoring the chemical reaction, the Au NBP-embedded metal nanoframes had better be treated as a whole, instead of the Au NBP and the nanoframe functioning separately as the nanoantenna for SERS and the catalytically active component, respectively.

## Conclusions

We have demonstrated the synthesis of plasmonic Au NBP-embedded ultrathin metal nanoframes through the site-selective deposition of metal atoms on Au NBPs. With the assistance of a silver layer that is pre-grown on the surface of Au NBPs, the supplied metal species can be directed to selectively nucleate and then grow with Ag at the edges and corners of the (Au NBP)@Ag nanorods in a pentagonal prism shape to form ultrathin metal nanoframes. The subsequent removal of Ag atoms results in the formation of Au NBP-embedded metal nanoframes. It is the unique geometrical structure rather than the composition of the deposited metals that results in the emergence of the interesting and attractive plasmonic properties for the Au NBP-embedded ultrathin metal nanoframes, as verified by FDTD simulations. The successful utilization of the facet-induced site-selective metal deposition method on (Au NBP)@Ag nanorods with different lengths and Au NBPs with different longitudinal dipolar plasmon wavelengths suggests the generality of the method. Moreover, the unique Au NBP-embedded metal nanoframes exhibit excellent SERS performances, making them feasible for *in situ* monitoring chemical reactions. Our work offers a versatile route for the synthesis of Au NBP-based ultrathin metal nanoframes with attractive plasmonic properties. These nanoframes will be useful for a variety of plasmon-based catalytic applications.

## Conflicts of interest

There are no conflicts to declare.

## Supplementary Material

SC-011-C9SC06475C-s001

## References

[cit1] Guo X., Ying Y. B., Tong L. M. (2014). Acc. Chem. Res..

[cit2] Schuller J. A., Barnard E. S., Cai W. S., Jun Y. C., White J. S., Brongersma M. L. (2010). Nat. Mater..

[cit3] Giannini V., Fernández-Dornínguez A. I., Heck S. C., Maier S. A. (2011). Chem. Rev..

[cit4] Atwater H. A., Polman A. (2010). Nat. Mater..

[cit5] Linic S., Aslam U., Boerigter C., Morabito M. (2015). Nat. Mater..

[cit6] Gawande M. B., Goswami A., Felpin F.-X., Asefa T., Huang X. X., Silva R., Zou X. X., Zboril R., Varma R. S. (2016). Chem. Rev..

[cit7] Mayer K. M., Hafner J. H. (2011). Chem. Rev..

[cit8] Saha K., Agasti S. S., Kim C., Li X. L., Rotello V. M. (2012). Chem. Rev..

[cit9] Tonga G. Y., Saha K., Rotello V. M. (2014). Adv. Mater..

[cit10] Yang X., Yang M. X., Pang B., Vara M., Xia Y. N. (2015). Chem. Rev..

[cit11] Ruan Q. F., Shao L., Shu Y. W., Wang J. F., Wu H. K. (2014). Adv. Opt. Mater..

[cit12] Ni W. H., Kou X. S., Yang Z., Wang J. F. (2008). ACS Nano.

[cit13] Ruditskiy A., Xia Y. N. (2016). J. Am. Chem. Soc..

[cit14] Sun X. J., Kim J. K., Gilroy K. D., Liu J. Y., König T. A. F., Qin D. (2016). ACS Nano.

[cit15] Niu Z. Q., Cui F., Kuttner E., Xie C. L., Chen H., Sun Y. C., Dehestani A., Schierle-Arndt K., Yang P. D. (2018). Nano Lett..

[cit16] Lin S.-C., Hsu C.-S., Chiu S.-Y., Liao T.-Y., Chen H. M. (2017). J. Am. Chem. Soc..

[cit17] Qin F., Zhao T., Jiang R. B., Jiang N. N., Ruan Q. F., Wang J. F., Sun L.-D., Yan C.-H., Lin H.-Q. (2016). Adv. Opt. Mater..

[cit18] Lu X. M., Au L., MaLellan J., Li Z.-Y., Marquez M., Xia Y. N. (2007). Nano Lett..

[cit19] Wan D. H., Xia X. H., Wang Y. C., Xia Y. N. (2013). Small.

[cit20] Greer J. R. (2014). Science.

[cit21] Liu Y. J., Pedireddy S., Lee Y. H., Hegde R. S., Tjiu W. W., Cui Y., Ling X. Y. (2014). Small.

[cit22] Zhang L., Liu T. Z., Liu K., Han L., Yin Y. D., Gao C. B. (2015). Nano Lett..

[cit23] Wang X., Ruditskiy A., Xia Y. N. (2016). Natl. Sci. Rev..

[cit24] Nosheen F., Zhang Z.-C., Zhuang J., Wang X. (2013). Nanoscale.

[cit25] Hu S., Wang X. (2013). Chem. Soc. Rev..

[cit26] Ye W., Kou S. F., Guo X., Xie F., Sun H. Y., Lu H. T., Yang J. (2015). Nanoscale.

[cit27] Hong X., Wang D. S., Cai S. F., Rong H. P., Li Y. D. (2012). J. Am. Chem. Soc..

[cit28] Chen C., Kang Y. J., Huo Z. Y., Zhu Z. W., Huang W. Y., Xin H. L., Snyder J. D., Li D. G., Herron J. A., Mavrikakis M., Chi M. F., More K. L., Li Y. D., Markovic N. M., Somorjai G. A., Yang P. D., Stamenkovic V. R. (2014). Science.

[cit29] Wu Y., Wang D. S., Zhou G., Yu R., Chen C., Li Y. D. (2014). J. Am. Chem. Soc..

[cit30] Fan N. N., Yang Y., Wang W. F., Zhang L. J., Chen W., Zou C., Huang S. M. (2012). ACS Nano.

[cit31] Xie S. F., Lu N., Xie Z. X., Wang J. G., Kim M. J., Xia Y. N. (2012). Angew. Chem., Int. Ed..

[cit32] Ren F. M., Wang Z., Luo L. F., Lu H. Y., Zhou G., Huang W. X., Hong X., Wu Y., Li Y. D. (2015). Chem.–Eur. J..

[cit33] Ye H. H., Wang Q. X., Catalano M., Lu N., Vermeylen J., Kim M. J., Liu Y. Z., Sun Y. G., Xia X. H. (2016). Nano Lett..

[cit34] McEachran M., Keogh D., Pietrobon B., Cathcart N., Gourevich I., Coombs N., Kitaev V. (2011). J. Am. Chem. Soc..

[cit35] Shahjamali M. M., Bosman M., Cao S. W., Huang X., Cao X. H., Zhang H., Pramana S. S., Xue C. (2013). Small.

[cit36] Sun X. J., Qin D. (2015). J. Mater. Chem. C.

[cit37] Ahn J., Wang D., Ding Y., Zhang J. W., Qin D. (2018). ACS Nano.

[cit38] Mahmoud M. A., El-Sayed M. A. (2010). J. Am. Chem. Soc..

[cit39] Deeb C., Zhou X., Plain J., Wiederrecht G. P., Bachelot R., Russell M., Jain P. K. (2013). J. Phys. Chem. C.

[cit40] Luo Y.-L., Shiao Y.-S., Huang Y.-F. (2011). ACS Nano.

[cit41] Li Q., Zhuo X. L., Li S., Ruan Q. F., Xu Q.-H., Wang J. F. (2015). Adv. Opt. Mater..

[cit42] Zhu X. Z., Zhuo X. L., Li Q., Yang Z., Wang J. F. (2016). Adv. Funct. Mater..

[cit43] Yang Y., Liu J. Y., Fu Z.-W., Qin D. (2014). J. Am. Chem. Soc..

[cit44] Sun X. J., Yang Y., Zhang Z. W., Qin D. (2017). Chem. Mater..

[cit45] Zhou M., Wang H. L., Vara M., Hood Z. D., Luo M., Yang T.-H., Bao S. X., Chi M. F., Xiao P., Zhang Y. H., Xia Y. N. (2016). J. Am. Chem. Soc..

[cit46] Gao C. B., Hu Y. X., Wang M. S., Chi M. F., Yin Y. D. (2014). J. Am. Chem. Soc..

[cit47] Itoh T., Yamamoto Y. S., Okamoto T. (2019). Phys. Rev. B.

[cit48] Zhu X. Z., Yip H. K., Zhuo X. L., Jiang R. B., Chen J. L., Zhu X. M., Yang Z., Wang J. F. (2017). J. Am. Chem. Soc..

[cit49] Habteyes T. G., Dhuey S., Cabrini S., Schuck P. J., Leone S. R. (2011). Nano Lett..

[cit50] Cui X. M., Qin F., Ruan Q. F., Zhuo X. L., Wang J. F. (2018). Adv. Funct. Mater..

[cit51] Prodan E., Radloff C., Halas N. J., Nordlander P. (2003). Science.

[cit52] Tsung C.-K., Kou X. S., Shi Q. H., Zhang J. P., Yeung M. H., Wang J. F., Stucky G. D. (2006). J. Am. Chem. Soc..

[cit53] Li J. M., Liu J. Y., Yang Y., Qin D. (2015). J. Am. Chem. Soc..

[cit54] Brus L. (2008). Acc. Chem. Res..

[cit55] Stiles P. L., Dieringer J. A., Shah N. C., Van Duyne R. P. (2008). Annu. Rev. Anal. Chem..

[cit56] Schlücker S. (2014). Angew. Chem., Int. Ed..

[cit57] McFarland A. D., Young M. A., Dieringer J. A., Van Duyne R. P. (2005). J. Phys. Chem. B.

[cit58] Huang J. F., Zhu Y. H., Lin M., Wang Q. X., Zhao L., Yang Y., Yao K. X., Han Y. (2013). J. Am. Chem. Soc..

[cit59] Xie W., Walkenfort B., Schlücker S. (2013). J. Am. Chem. Soc..

[cit60] Xie W., Herrmann C., Kömpe K., Haase M., Schlücker S. (2011). J. Am. Chem. Soc..

[cit61] Zhang J. W., Winget S. A., Wu Y. R., Su D., Sun X. J., Xie Z.-X., Qin D. (2016). ACS Nano.

