# Supplementary material for: Gold nanobipyramid-embedded ultrathin metal nanoframes for in situ monitoring catalytic reactions
Source: Chem Sci. 2020 Feb 26;11(12):3198–207. doi: 10.1039/c9sc06475c (PMC8157342; doi:10.1039/c9sc06475c)
Supplement: SC-011-C9SC06475C-s001 [file SC-011-C9SC06475C-s001.pdf]

Electronic Supplementary Material (ESI) for Chemical Science.
This journal is © The Royal Society of Chemistry 2020

Electronic supplementary information

Gold nanobipyramid-embedded ultrathin metal nanoframes for *in situ* monitoring catalytic reactions

Xingzhong Zhu,[a] Juan Xu,[a] Han Zhang,[b] Ximin Cui,[b] Yanzhen Guo,[b] Si Cheng,[c] Caixia Kan*[a] and Jianfang Wang*[b]*

[a]College of Science, Nanjing University of Aeronautics and Astronautics, Nanjing 210016, China

[b]Department of Physics, The Chinese University of Hong Kong, Shatin, Hong Kong SAR, China

[c]College of Chemistry, Chemical Engineering and Materials Science, Soochow University, Suzhou 215021, China

Experimental

Chemicals

$HAuCl_4 \cdot 3H_2O$ (99%), $PdCl_2$ (99%), H_2PtCl_6 (99%), $NaBH_4$ (98%), trisodium citrate (99%), ascorbic acid (99%) and $AgNO_3$ (99%) were purchased from Sigma-Aldrich. CTAB (98%) were obtained from Alfa Aesar. H_2O_2 solution (30 wt% in water), cetyltrimethylammonium chloride (CTAC, 97%), NaOH (96%), $NH_3 \cdot H_2O$ solution (25 wt% in water) and HCl solution (37 wt% in water) were purchased from Aladdin Reagent. Deionized water with a resistivity of 18.2 MΩ cm produced by a Direct-Q 5 ultraviolet water purification system was used in all experiments.

Synthesis of the Au NBPs

The Au NBP samples were grown through seed-mediated growth in aqueous solutions, as described in previous works. Briefly, a freshly prepared, ice-cold $NaBH_4$ solution (0.15 mL, 0.01 M) was injected quickly into an aqueous solution that was pre-made by mixing together $HAuCl_4$ (0.125 mL, 0.01 M), trisodium citrate (0.25 mL, 0.01 M) and water (9.625 mL). The resultant seed solution was kept at room temperature for 2 h before use. The seed solution (0.26 mL) was injected into the growth solution that was made in advance by mixing together CTAB (40 mL, 0.1 M), $HAuCl_4$ (2 mL, 0.01 M), $AgNO_3$ (0.4 mL, 0.01 M), HCl (0.8 mL, 1 M) and ascorbic acid (0.32 mL, 0.1 M), followed by gentle inversion mixing for 10 s. The reaction solution was left undisturbed overnight at room temperature. The longitudinal dipolar plasmon wavelength of the obtained Au NBP sample was 794 nm. The amounts of the added seed solution were 2 mL, 0.2 mL, and 0.095 mL for the growth of the 679-, 906-, and 979-nm Au NBP samples, respectively. For Au NBPs with longer longitudinal dipolar plasmon wavelengths, a cetyltributylammonium bromide (CTBAB) growth solution was prepared by the sequential addition of $HAuCl_4$ (1.2 mL, 0.01 M), $AgNO_3$ (0.6 mL, 0.01 M) and ascorbic acid (0.4 mL, 0.1 M) into an aqueous CTBAB solution (28.5 mL, 0.01 M). The seed solution was then added. The reaction solution was mixed by gentle inversion for 10 s and left undisturbed overnight in an oven at 60 °C. The amount of the added seed solution was 0.063 mL for the growth of the 1074-nm Au NBP sample. The purification of the as-prepared Au NBPs was conducted using a depletion-induced separation method. The number percentage of the purified Au NBPs was found from TEM imaging to be 99%.

Synthesis of the ultrathin metal nanoframes

The Au NBP-embedded ultrathin metal nanoframes with different compositions and shapes were produced following the same procedure, with the amount of the involved metal precursor varied. In a typical synthesis, the purified Au NBPs (10 mL, the longitudinal dipolar plasmon peak extinction value was adjusted to ~1.0 using a 0.5-cm cuvette) were centrifuged at 7000 rpm for 10 min. The precipitate was redispersed into a CTAC solution (10 mL, 0.08 M), followed by the subsequent addition and mixing of $AgNO_3$ (250 µL, 0.01 M) and ascorbic acid (125 µL, 0.1 M) under gentle shaking. The mixture solution was placed in an air-bath shaker (65 °C, 100 revolutions per minute) and kept for 4.5 h, during which Ag was overgrown on the Au NBPs to form (Au NBP)@Ag nanorods. The resultant sample was centrifuged twice at 6000 rpm for 10

min. The precipitate was redispersed into a CTAB solution (4 mL, 0.05 M). The formation of the metal nanoframes on the surfaces of the (Au NBP)@Ag nanostructures was carried out by the sequential addition of NaOH (1 mL, 0.2 M) and ascorbic acid (1 mL, 0.1 M) under gentle shaking. An aqueous $HAuCl_4$ (0.1 mM) solution was then added into the solution using a syringe pump at a rate of 20 μL min^{-1} under ambient conditions. The volume of the $HAuCl_4$ solution was varied from 0.4 mL to 3.2 mL. After the addition was completed, the reaction was left for another 20 min. The resultant sample was centrifuged twice at 6000 rpm for 10 min. The precipitate was redispersed into water (6 mL), followed by the addition of CTAB (0.3 mL, 0.1 M), H_2O_2 (4 mL, 6 wt%) and $NH_3 \cdot H_2O$ (0.2 mL, 28 wt%). The resultant sample was left at room temperature for 12 h and then centrifuged at 3500–5000 rpm for 10 min. The precipitate was redispersed into water (5 mL) for further use. The preparation of the ultrathin Pd and Pt nanoframes encapsulating the Au NBPs followed the same procedure as that for the Au NBP-embedded Au nanoframes except that $HAuCl_4$ was replaced with H_2PdCl_4 and H_2PtCl_6, respectively.

Synthesis of the (Au NBP)/Pd and (Au NBP)/Pt nanostructures

The deposition of Pd and Pt atoms on the Au NBPs followed previous works with minor modification. Typically, the purified Au NBPs (10 mL, the longitudinal dipolar plasmon peak extinction value was adjusted to ~1.0 using a 0.5-cm cuvette) were centrifuged at 7000 rpm for 10 min. The precipitate was redispersed into a CTAB solution (10 mL, 0.025 M), followed by subsequent addition of H_2PdCl_4 (180 μL, 1 mM) and ascorbic acid (90 μL, 0.01 M) under gentle shaking. The reaction was left undisturbed overnight at room temperature. The resultant sample was centrifuged at 5500 rpm for 10 min. The precipitate was redispersed into water (5 mL) for further use. The preparation of the (Au NBP)/Pt nanostructures followed the same procedure as that of the (Au NBP)/Pd nanostructures, with H_2PdCl_4 replaced by H_2PtCl_6 and room temperature changed to 60 °C.

SERS monitoring of the reaction

The reduction of 4-NTP on the Au NBP-embedded ultrathin metal nanoframes was employed as a model reaction. The ultrathin metal nanoframes (0.9 mL) were mixed with an ethanolic 4-NTP solution (0.1 mL, 10^{-4} M) and incubated at room temperature for 12 h. The 4-NTP-

functionalized nanoframes were then washed with water twice and re-suspended in water (1 mL). The suspension (0.4 mL) of the 4-NTP-adsorbed nanoframes was mixed with an aqueous $NaBH_4$ (0.6 mL, 1 mM) solution to start the reaction at room temperature. The SERS spectra were recorded at 2-min intervals directly from the solution under 785-nm laser excitation at a laser power of 6.3 W cm^{-2}.

Instrumentation

Extinction spectra were measured on a Shimadzu UV-3600 Plus ultraviolet/visible/near-infrared spectrophotometer with plastic cuvettes of 0.5-cm optical path length. For the measurements beyond 1400 nm, water was replaced by deuterium oxide. The samples were centrifuged and washed twice by deuterium oxide and then redispersed in deuterium oxide. The cuvettes with 1-mm optical path length were used to reduce the overall extinction. TEM imaging was carried out on an FEI Tecnai 12 microscope operated at 120 kV. HRTEM imaging, HAADF-STEM characterization and elemental mapping were performed on an FEI Tecnai F20 microscope operated at 200 kV and equipped with an Oxford energy-dispersive X-ray (EDX) analysis system. ICP-OES measurements were performed on an Agilent ICP-MS 7500a system. Raman spectra were recorded on a Horiba Jobin Yvon LabRAM HR-800 spectrophotometer with 514-nm, 633-nm and 785-nm laser excitation.

FDTD simulations

FDTD simulations were performed using FDTD Solutions 8.7 (Lumerical Solutions). During the simulations, an electromagnetic pulse in the spectral range from 300 nm to 5000 nm was launched into a box containing a target nanostructure. A mesh size of 0.5 nm was employed in calculating the extinction spectra and charge distribution contours of the Au NBP-embedded Au, Ag, Pd and Pt nanoframes. The refractive index of the surrounding medium was set at 1.33, that of water. The dielectric function of Au was obtained by fitting the measured data of Johnson and Christy, and those of Ag, Pd and Pt were fitted from Palik's data. The sizes of the Au NBPs and the NBP-based nanostructures were set according to the average waist widths and lengths measured from the TEM images. The waist width and length of the Au NBP were set at 31 nm and 98 nm, respectively. The two ends of the Au NBP were approximated as two pentapyramids with the height and base width (distance from one apex to the opposite pentagonal edge) adjusted

to be 7 nm and 8 nm, respectively. The diameter and length of the (Au NBP)@Ag nanorod were set at 32 nm and 135 nm, respectively. The thickness and length of the ridge of the nanoframe was 2 nm and 119 nm, respectively, with the tip height adjusted to be 9 nm. The nanoframe was in direct contact with the waist corners of the Au NBP. The length of the nanoframe was increased from 120 nm to 250 nm. Because we were interested in the variation behaviors of the longitudinal plasmon peak position, the excitation light direction was set to be perpendicular to the length axis, with the electric field aligned along the length axis.

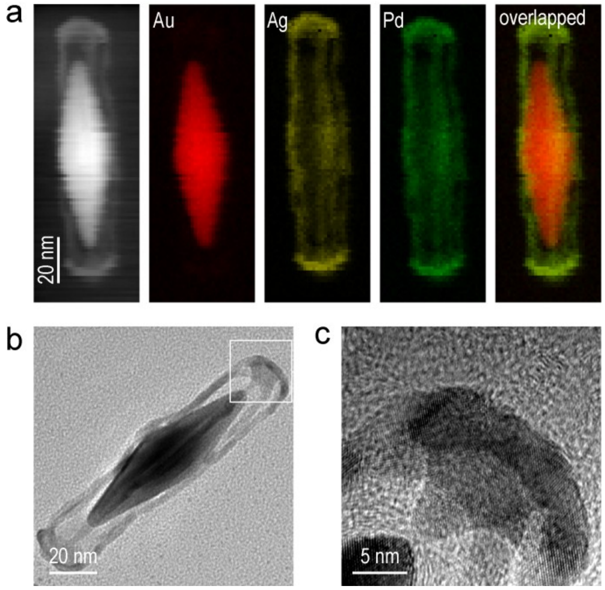

Fig. S1 Au NBP-embedded ultrathin Pd nanoframes. (a) HAADF-STEM and elemental mapping images of a single nanoframe. The elemental mapping images have the same scale bars as the HAADF-STEM image. (b) TEM image of a single nanoframe. (c) HRTEM image recorded in the region indicated with the white box in (b).

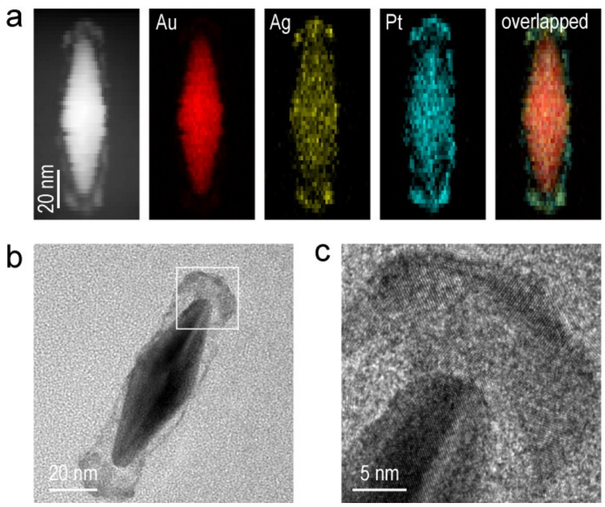

Fig. S2 Au NBP-embedded ultrathin Pt nanoframes. (a) HAADF-STEM and elemental mapping images of a single nanoframe. The elemental mapping images have the same scale bars as the HAADF-STEM image. (b) TEM image of a single nanoframe. (c) HRTEM image recorded in the region indicated with the white box in (b).

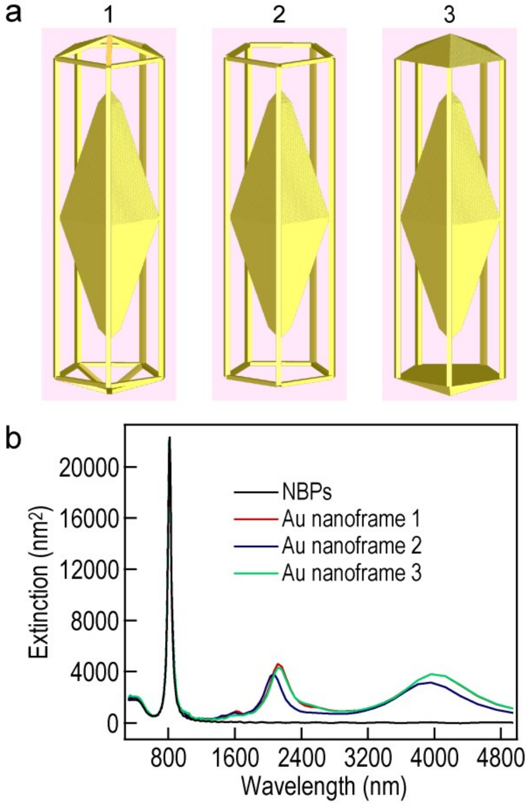

Fig. S3 FDTD simulations. (a) Schematics of the models used in the simulations. (b) Simulated extinction spectra of the Au NBP-embedded Au nanoframes 1, 2 and 3.

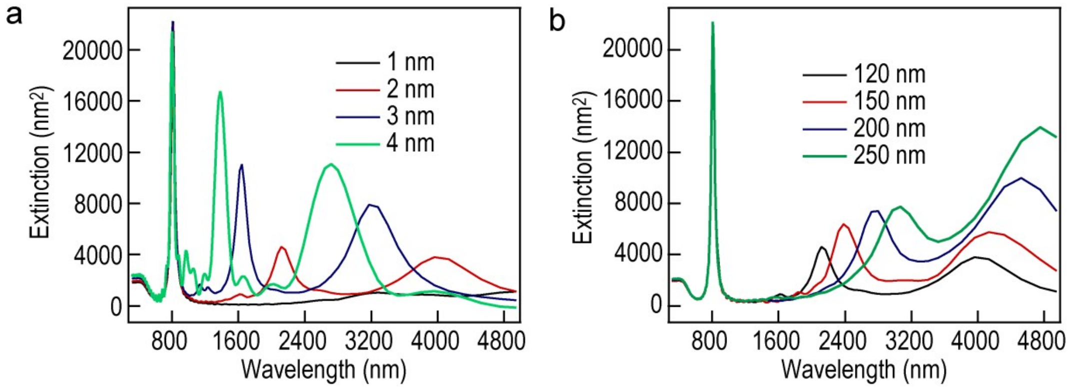

Fig. S4 FDTD simulations. (a) Simulated extinction spectra of the Au NBP-embedded Au nanoframes with the ridges having different widths, as indicated in the inset. (b) Simulated extinction spectra of the Au NBP-embedded Au nanoframes with the side ridges having different lengths, as indicated in the inset. The excitation polarization direction is along the length axis of the Au nanoframe.

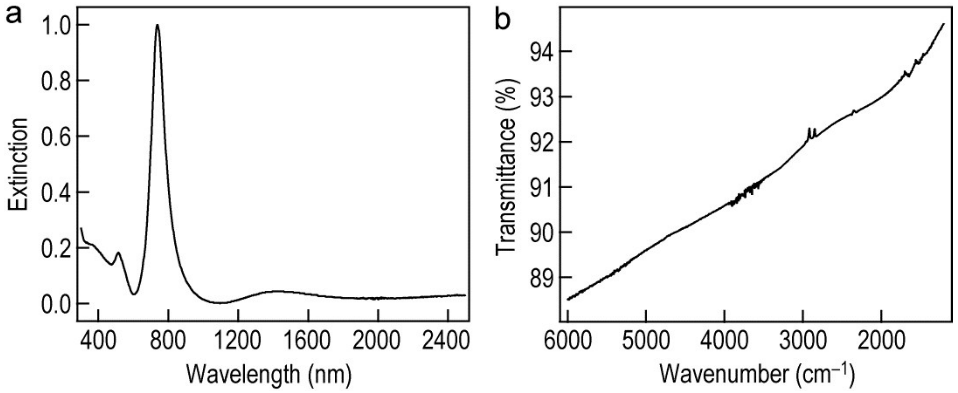

Fig. S5 Extinction and transmittance spectra of the Au NBP-embedded ultrathin Au nanoframe sample. (a) Extinction spectrum measured in the spectral range from 300 nm to 2500 nm. (b) Transmittance spectrum in the mid-infrared region.

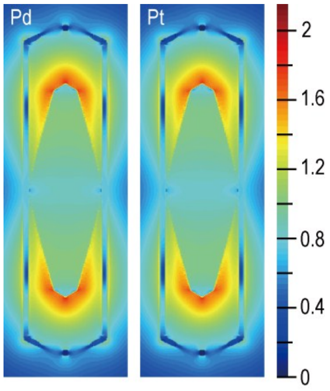

Fig. S6 Simulated electric field enhancement contours under the longitudinal excitation at 815 nm for the Au NBP-embedded Pd (left) and Pt (right) nanoframes, respectively. The field enhancement contours are drawn at the logarithmic scale.

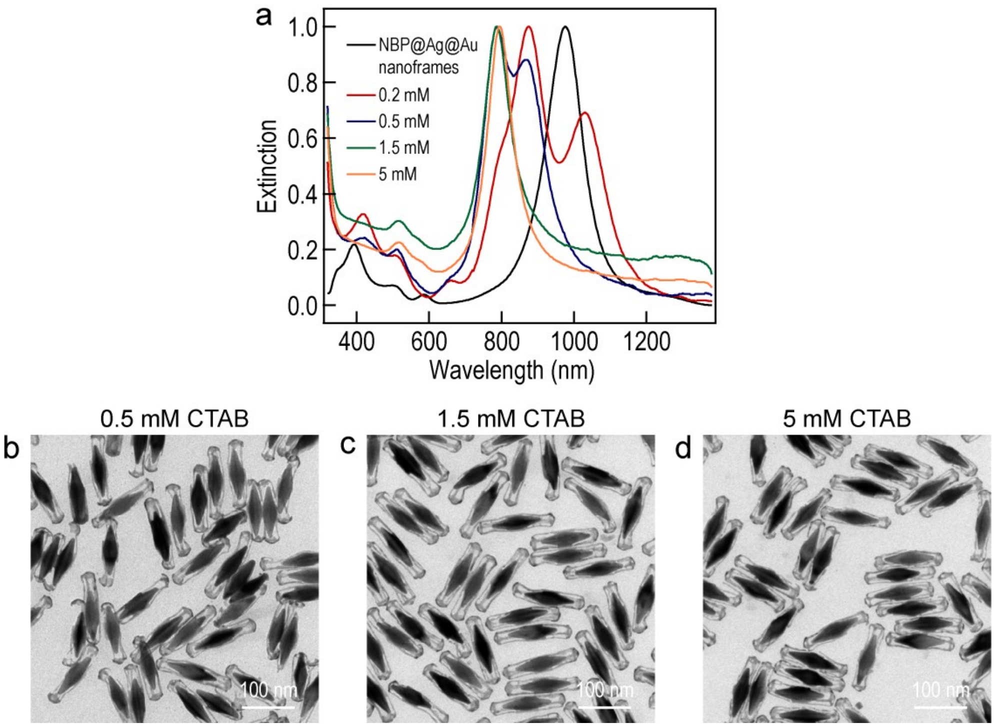

Fig. S7 Effect of the CTAB concentration on the Ag etching process. The supplied volumes of $AgNO_3$ (0.01 M) and $HAuCl_4$ (0.1 mM) are 250 μL and 1.4 mL, respectively. (a) Normalized extinction spectra of the products obtained by etching the (Au NBP)@Ag@Au nanoframes in CTAB solutions at different concentrations. (b–d) TEM images of the products obtained in the CTAB solutions at 0.5 mM, 1.5 mM and 5 mM, respectively.

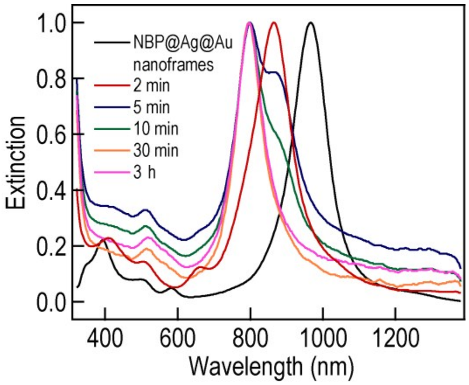

Fig. S8 Normalized extinction spectra of the products collected at different time points after the addition of $NH_3{\cdot}H_2O$ and H_2O_2 during the Ag etching process. The supplied volumes of $AgNO_3$ (0.01 M) and $HAuCl_4$ (0.1 mM) are 250 μL and 1.4 mL, respectively.

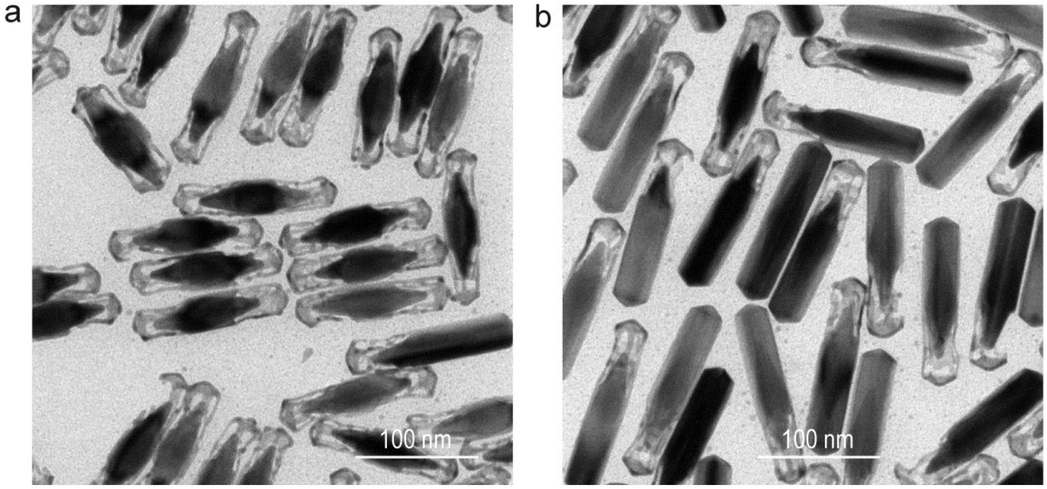

Fig. S9 Higher-magnification TEM images of the produced Au NBP-embedded nanostructure samples. (a) For the image shown in Fig. 3g. (b) For the image shown in Fig. 3h.

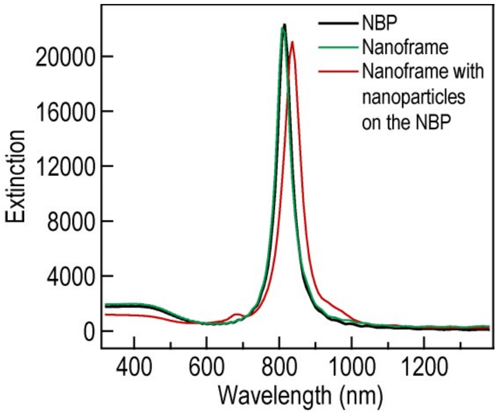

Fig. S10 Simulated extinction spectra of the Au NBP and the Au NBP-embedded Au nanoframe with some Au nanoparticles randomly deposited on the edges and flat facets of the Au NBP. The diameter of the Au nanoparticles is 6 nm.

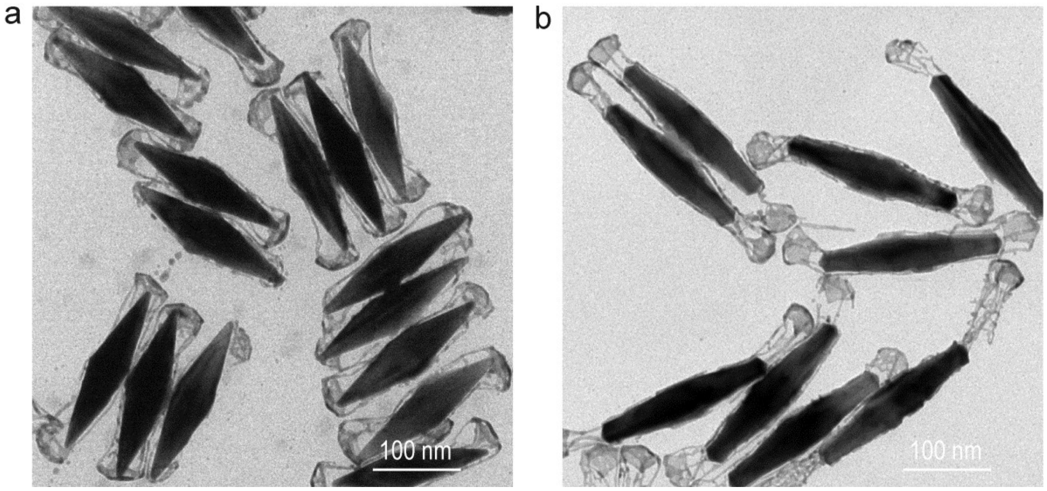

Fig. S11 TEM image of the ultrathin Au nanoframes grown with more $HAuCl_4$. (a) Produced from the sample 4 in Fig. 5b with the volume of $HAuCl_4$ (0.1 mM) being 2.2 mL. (b) Produced from the sample 5 in Fig. 5b with the volume of $HAuCl_4$ (0.1 mM) being 3.1 mL.

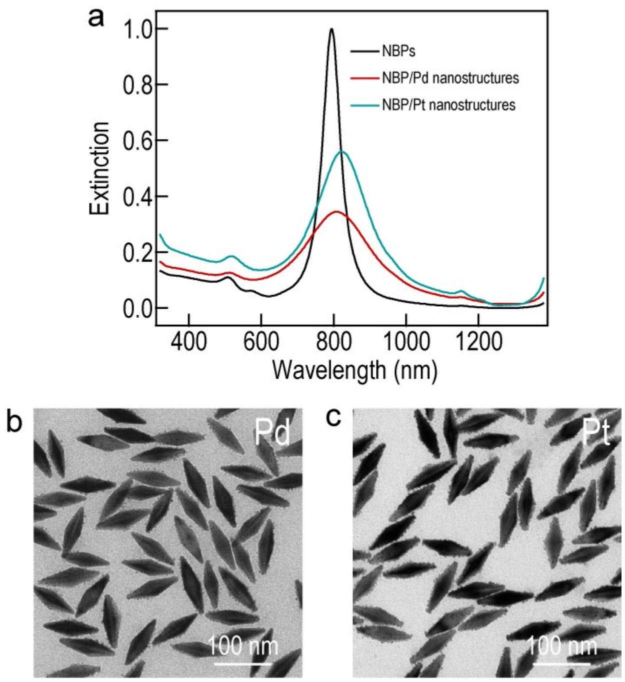

Fig. S12 (Au NBP)/Pd and (Au NBP)/Pt nanostructures. (a) Extinction spectra of the nanostructure samples. (b) TEM image of the (Au NBP)/Pd nanostructures. c) TEM image of the (Au NBP)/Pt nanostructures.

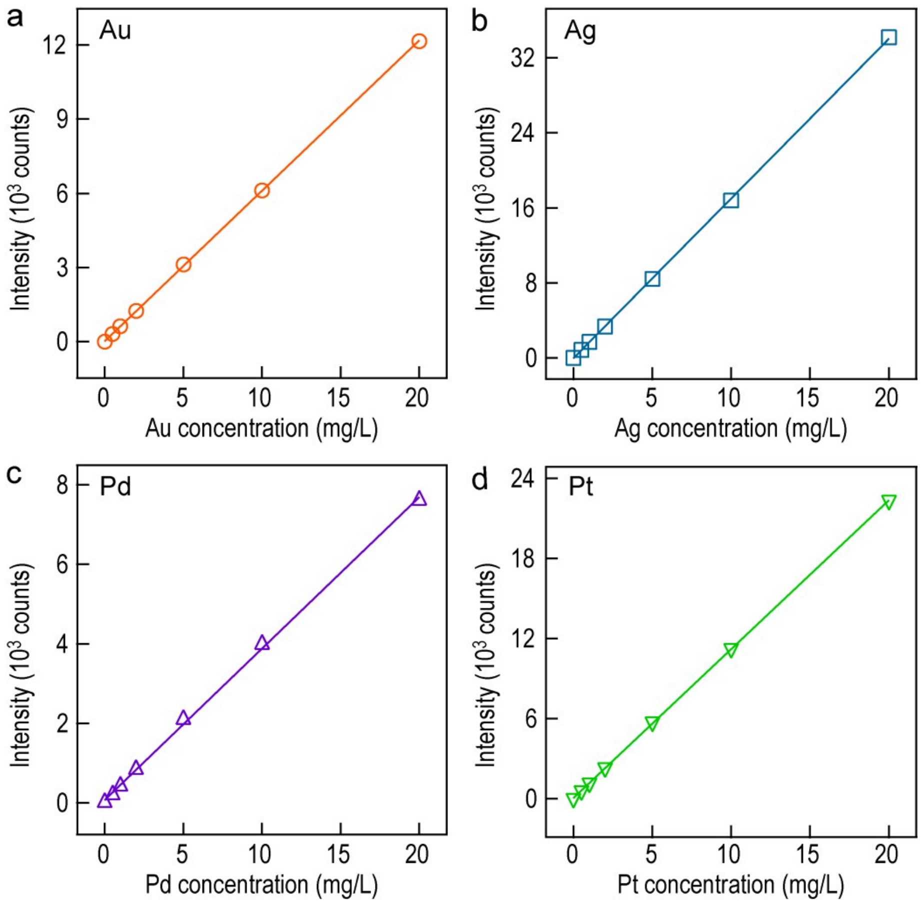

Fig. S13 Linear relationships between the emission intensity and the atomic mass concentration for the ICP-OES measurements. The markers represent the atomic mass concentrations of 0, 0.5, 1, 2, 5, 10 and 20 mg L^{-1}, respectively. (a) For Au. The equation obtained from linear fitting is $y = (608 \pm 2)\,x + (19 \pm 14)$. The coefficient of determination is $R^2 = 0.9999$. (b) For Ag. The linear equation is $y = (1705 \pm 6)\,x - (51 \pm 55)$. The coefficient of determination is $R^2 = 0.9999$. (c) For Pd. The linear equation is $y = (381 \pm 5)\,x + (73 \pm 44)$. The coefficient of determination is $R^2 = 0.9991$. (d) For Pt. The linear equation is $y = (1116 \pm 3)\,x + (46 \pm 27)$. The coefficient of determination is $R^2 = 0.9999$.

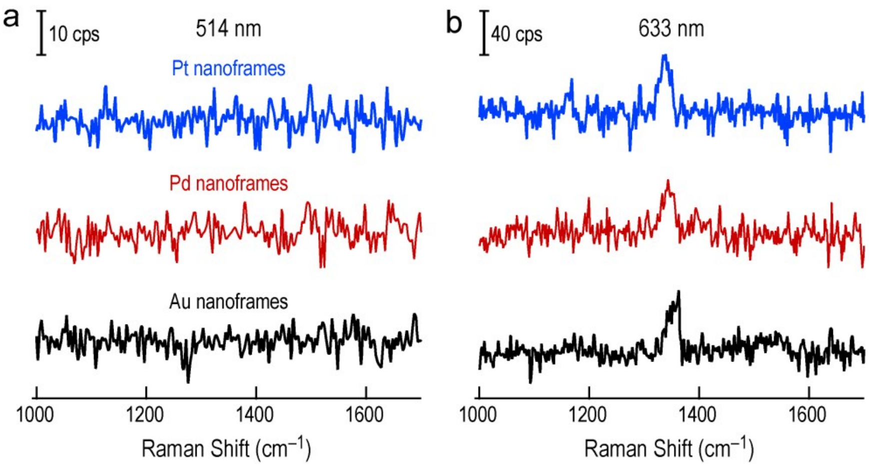

Fig. S14 SERS measurements. (a) SERS spectra collected from 4-NTP adsorbed on the ultrathin Au, Pd and Pt nanoframes under excitation at 514 nm. (b) SERS spectra of the three samples collected similarly under excitation at 633 nm.

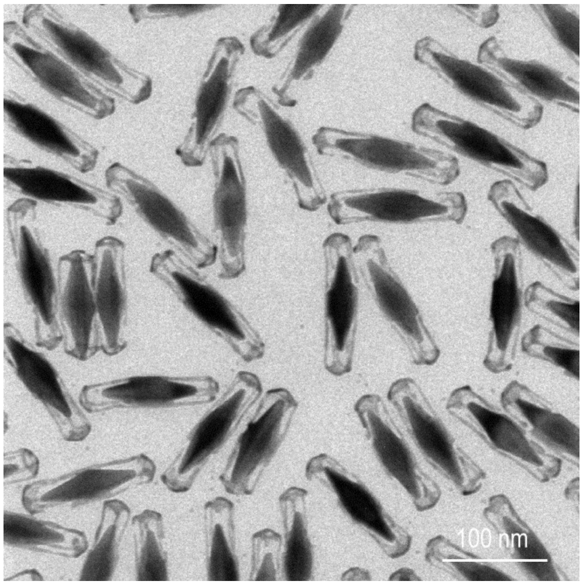

Fig. S15 TEM image of the ultrathin Au nanoframes after the *in situ* monitoring of the catalytic reaction.

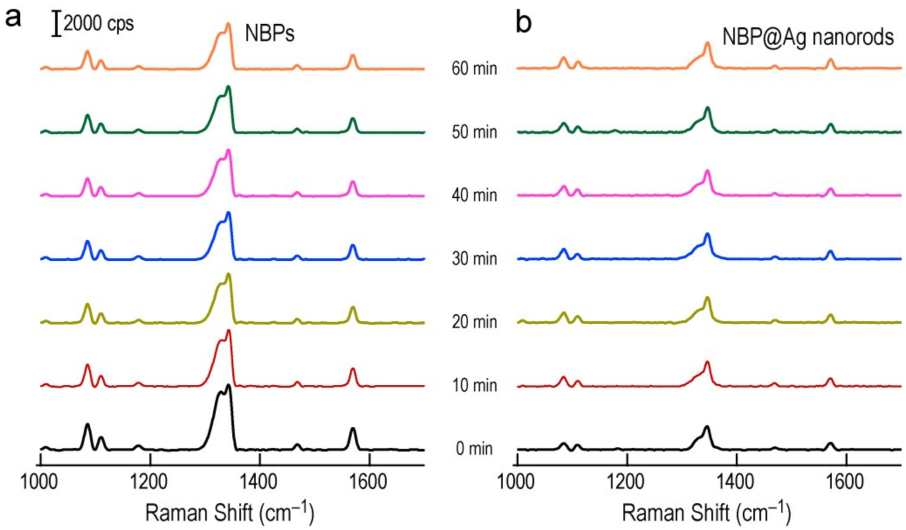

Fig. S16 SERS measurements. (a) SERS spectra recorded with the Au NBPs as the catalyst. (b) SERS spectra recorded with the (Au NBP)@Ag nanorods as the catalyst. The SERS spectra were collected as functions of time during the reduction of 4-NTP by $NaBH_4$ under excitation at 785 nm.

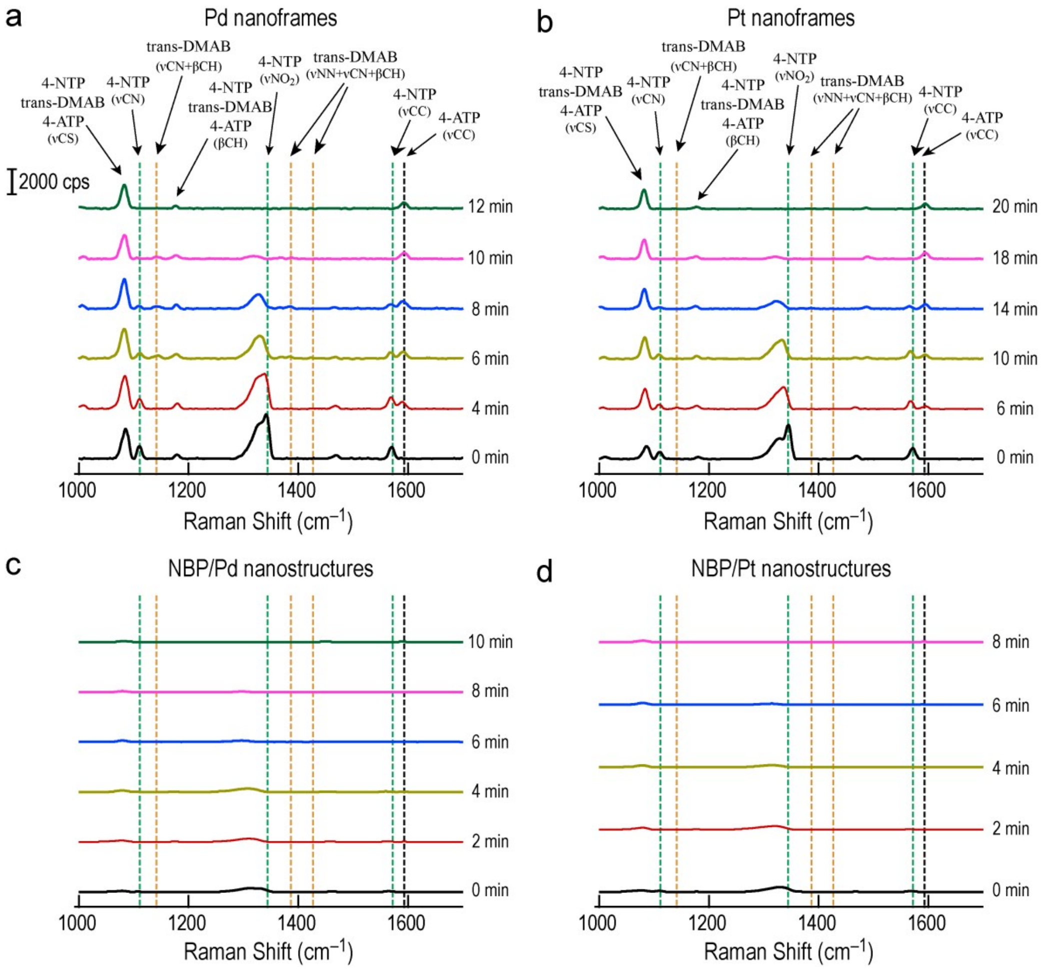

Fig. S17 SERS measurements. (a–d) Time-dependent SERS spectra collected during the reduction of 4-NTP by $NaBH_4$ under excitation at 785 nm. The reaction was catalyzed by the Au NBP-embedded Pd, Pt nanoframes and the (Au NBP)/(Pd, Pt) nanostructures, respectively.

Table S1 Au, Ag, Pd and Pt mass concentrations in the Au NBP, Au NBP-embedded ultrathin metal (Au, Pd, Pt) nanoframe, and (Au NBP)/(Pd, Pt) nanostructure samples measured by ICP-OES

Sample	Au (μg mL^{-1})	Ag (μg mL^{-1})	Pd (μg mL^{-1})	Pt (μg mL^{-1})
Au NBPs	7.0	0.05		
Au nanoframes	8.1	0.55		
Pd nanoframes	7.0	0.56	0.41	
Pt nanoframes	6.8	0.68		0.89
NBP/Pd nanostructures	6.9		0.40	
NBP/Pt nanostructures	6.8			0.92

Table S2 Values of N_{surf}, I_{SERS}, N_{vol}, and I_{NRS} for the Au NBP, Au NBP-embedded ultrathin metal (Au, Pd, Pt) nanoframe, and (Au NBP)/(Pd, Pt) nanostructure samples[a]

<table>
<tr><th>Sample</th><th>N_{surf} (10^{16})</th><th>I_{SERS}</th><th>N_{vol} (10^{22})</th><th>I_{NRS}</th></tr>
<tr><td>Au NBPs</td><td>2.27</td><td>27054</td><td rowspan="6">6</td><td rowspan="6">588</td></tr>
<tr><td>Au nanoframes</td><td>5.16</td><td>16392</td></tr>
<tr><td>Pd nanoframes</td><td>5.14</td><td>12928</td></tr>
<tr><td>Pt nanoframes</td><td>5.34</td><td>12698</td></tr>
<tr><td>NBP/Pd nanostructures</td><td>3.01</td><td>753</td></tr>
<tr><td>NBP/Pt nanostructures</td><td>3.51</td><td>2149</td></tr>
</table>

[a] N_{surf} refers to the number of 4-NTP molecules bound to the enhancing metal nanostructures. I_{SERS} is the surface-enhanced Raman intensity. N_{vol} represents the number of the molecules in the excitation volume. I_{NRS} is the normal Raman intensity.
